# Colloids as Mobile Substrates for the Implantation and Integration of Differentiated Neurons into the Mammalian Brain

**DOI:** 10.1371/journal.pone.0030293

**Published:** 2012-01-25

**Authors:** Dennis Jgamadze, Jamie Bergen, Daniel Stone, Jae-Hyung Jang, David V. Schaffer, Ehud Y. Isacoff, Sophie Pautot

**Affiliations:** 1 Center for Regenerative Therapies Dresden, Dresden, Germany; 2 Department of Molecular and Cell Biology, University of California, Berkeley, Physical Biosciences Division, Lawrence Berkeley National Laboratory, Berkeley, California, United States of America; 3 Department of Chemical Engineering, University of California, Berkeley, California, United States of America; University of California, Berkeley, United States of America

## Abstract

Neuronal degeneration and the deterioration of neuronal communication lie at the origin of many neuronal disorders, and there have been major efforts to develop cell replacement therapies for treating such diseases. One challenge, however, is that differentiated cells are challenging to transplant due to their sensitivity both to being uprooted from their cell culture growth support and to shear forces inherent in the implantation process. Here, we describe an approach to address these problems. We demonstrate that rat hippocampal neurons can be grown on colloidal particles or beads, matured and even transfected *in vitro*, and subsequently transplanted while adhered to the beads into the young adult rat hippocampus. The transplanted cells have a 76% cell survival rate one week post-surgery. At this time, most transplanted neurons have left their beads and elaborated long processes, similar to the host neurons. Additionally, the transplanted cells distribute uniformly across the host hippocampus. Expression of a fluorescent protein and the light-gated glutamate receptor in the transplanted neurons enabled them to be driven to fire by remote optical control. At 1-2 weeks after transplantation, calcium imaging of host brain slice shows that optical excitation of the transplanted neurons elicits activity in nearby host neurons, indicating the formation of functional transplant-host synaptic connections. After 6 months, the transplanted cell survival and overall cell distribution remained unchanged, suggesting that cells are functionally integrated. This approach, which could be extended to other cell classes such as neural stem cells and other regions of the brain, offers promising prospects for neuronal circuit repair via transplantation of *in vitro* differentiated, genetically engineered neurons.

## Introduction

Dysfunctions in synaptic transmission and degeneration of specific classes of neurons are at the origin of many neurological disorders [1], [2], [3], [4], [5], [6], [7], [8]. The limited capacity of the mammalian central nervous system for self-repair makes cell transplantation an attractive approach to replace cells in damaged areas of the brain. The early signs of success of neural tissue grafts in animal models for disorders such as stroke [9], [10], Huntington's disease [11], brain lesion [12], and Parkinson disease [13], [14] have made cell replacement therapy a highly promising clinical approach. However, in some cases, tissue grafts lead to an inflammatory response and problems with deep tissue innervation suggesting that dissociated neurons may be more effective. Several sources of dissociated neurons have been considered for replacement therapy. Embryonic neurons can better recover from dissociation than fully mature neurons, and they can subsequently differentiate into mature neurons, making them a promising source for cell therapies. Nonetheless, to preserve a good viability, these cells have to be harvested at a very specific embryonic stage and transplanted immediately after dissociation [15]. Homotopic transplantation of normal embryonic neurons into the striatum of Huntington's disease and Parkinson disease animal models [16], [17], [18], and into the hippocampus in models of temporal lobe epilepsy [19], appear to lead to cell survival and functional integration. However, the transplanted neurons remain within the injection area, limiting the reach of the functional repair.

The emergence of multipotent or pluripotent stem cells has provided expandable sources of cells that can be manipulated, differentiated in culture and subsequently transplanted [20], [21], [22], [23], [24], [25], [26], [27], [28], [29], [30]. Transplanted neuronal progenitor cells can show good survival after injection and exhibit the ability to migrate away from the injection location; however only a small fraction of the transplanted cells become committed to a neuronal fate, and the cells retain the potential to generate tumors [31]. As a result, great effort has been spent to make neural stem cells (NSCs) commit to a differentiated post-mitotic state prior to transplantation [24], [32], [33].

Regardless of the progresses made to derive the major brain cell types from stem cells [28], [30], [32], [34], [35] the major challenge remains to bypass the dissociation step to harvest and transplant the differentiated cells without damaging them and in a form that permits integration *in vivo*.

We have shown that primary neurons can be grown and differentiated on beads where they are amenable to transfection or viral infection just as they are on a conventional flat substrate [36]. Importantly, the bead-supported neurons can be readily moved without damage to the cells, avoiding the problem of breaking the cells away from their growth surface. Here we ask whether bead-borne neurons can be injected into a recipient brain for transplantation. We focus on the uninjured hippocampus and show that differentiated pre- and post-natal rat hippocampal neurons on beads, transduced with genes encoding a fluorescent protein and a light-gated excitatory ion channel, can be homotopically transplanted into a young adult rat hippocampus. The transplanted neurons leave the beads, disperse from the site of injection, and exhibit high survival rates. Furthermore, the transplanted neurons subsequently form functional connections with the host neurons, as demonstrated by calcium imaging during optical stimulation of the transplanted cells. After 6 months, the transplanted animals were healthy and seizure free. No significant changes in cell survival and cell distribution was observed. Thus this approach could potentially facilitate the replacement of degenerated neurons in older subjects with fully differentiated neurons obtained from either embryonic culture or *in vitro* differentiation of NSCs or induced pluripotent stem cells. This method holds the promise of two additional advantages that come with the ability to sort the beads prior to transplantation: pre-selection of cells that are healthy and that are in a specific differentiated state.

## Results

**Figure 1:**
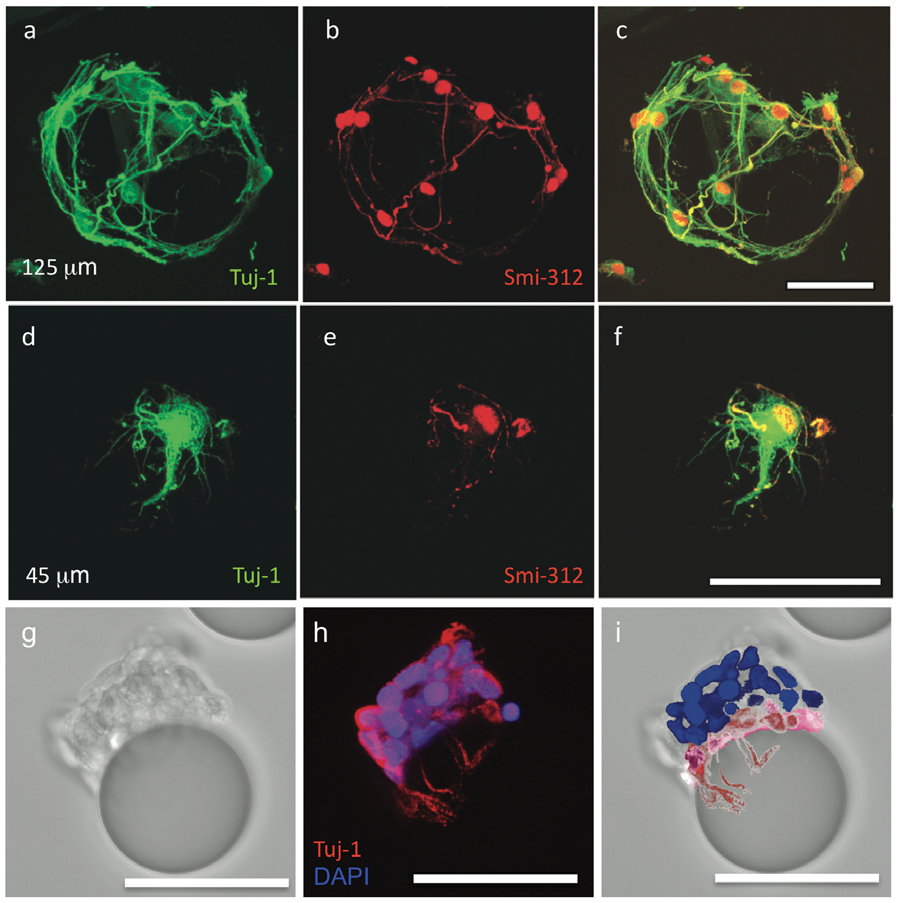
Development and manipulation of neurons supported on silica beads. Confocal microscopy z series are projected on the xy scanning plane. E18 hippocampal neurons cultured seeded at 4k cells/cm^2^ on 125 µm (**a-c**), and on 45 µm (**d-f**) PLL coated beads shown at DIV 4. Cells were fixed and stained with a neuron specific alphãtubulin antibody (green), and with an axon specific smi-312 antibody (red). Neurons were polarized in both preparations independently of bead radius of curvature. The number of neurons per bead is proportional to bead surface area, as 45 µm beads carried on average one cell, and 125 µm beads carried about 10 cells. (**g**) Bright field image of neurons seeded at 100k cells/cm^2^ on 45 µm beads at DIV 4. (**h**) Cells were fixed and stained with a neuron specific Tuj-1 antibody (red), and the nuclear marker DAPI (blue). Twenty-one of the twenty-five cells on this bead are Tuj-1 positive. At this high density, cells in direct contact with the bead surface wrap their processes around the beads (highlighted in red) while the others sit on this layer (highlighted in blue) as illustrated in the color-coded picture (**i**). All Scale bars  =  50 µm.

The success of neuronal transplantation depends on the composition [15], [37] and the health of the injected cells [15], as well as on the level of trauma created by the transplantation procedure. To obtain a neuron-rich, *in vitro* culture we employed rat hippocampal neurons. Late embryonic stage (E18) hippocampal neurons were seeded on poly-L-lysine (PLL) coated beads using standard techniques developed for conventional 2D cultures [38], [39] and adapted for 3D supports [36]. At day 3 *in vitro* (DIV 3), 125 µm beads were primarily populated by neurons (∼90% were Tuj-1 positive), and process branching was comparable to that of 2D cultures (**Fig. 1a–c**). Young hippocampal cultures are poor in glia cells, hence we restored the glial growth factors known to contribute to neuronal development [40] with conditioned media from glial feeder cell cultures. As a result we observed a robust growth of mature neurons even at the low cell surface density of 4k cells/cm^2^.

### Carrier bead optimization for neuron transplantation

Injecting solid material into a soft-tissue such as the brain can lead to a transient increase in pressure, limiting the quantity of material that can be transplanted. Hence, to minimize the carrier volume while maximizing the number of injected cells, we chose 45 µm diameter beads, the smallest size offering a surface large enough to accommodate neuronal processes over a week while enabling growth of the largest number of cells per volume [36]. Immuno-staining for smi-312, an axonal marker, revealed that the E18 neurons at DIV 4 were polarized on the smaller beads just as well as they were on the larger ones even at the low cell density of 4k cells/cm^2^ (**Fig. 1d–f**), confirming that this smaller bead size does not compromise the neuron maturation process. Although initially confined to their carrier beads, neurons can bridge to the surface accessible in its surrounding beads to seek new cognate partners and form a high density of synapses across the space (see **Supplementary Fig. S1**), suggesting that these cells have maintained a high connectivity potential.

To maximize the number of cells injected, we increased the cell seeding density to 100k cells/cm^2^ (**Fig. 1–g–i**) to obtain 20 to 50 cells per 45 µm bead. Due to the limited area of the 45 µm beads a small number of the cells made direct contact with the bead surface and the remaining cells rested on those. Prior to injection when the beads were still in culture, the dendritic and axonal arbors were complex for the cells that directly contacted the bead but less developed for cells adhering to other cells. We determined that, at the time of injection, the neuron on bead culture was composed of 70.4% of NeuN positive cells, a nuclear neurons marker, 13.2% of GFAP positive cells, an astrocyte cell marker, and 16.1% of nestin positive cells, as well as 5.6% positive cells for Musashi, and 4.8% are positive cells for Sox2 (see **Supplementary Fig. S2**). We also determined that 10.7% of the NeuN positive cells are positive for GAD67 an inhibitory neuronal cell marker.

### Neuronal transplantation in the young adult rat hippocampus

**Figure 2:**
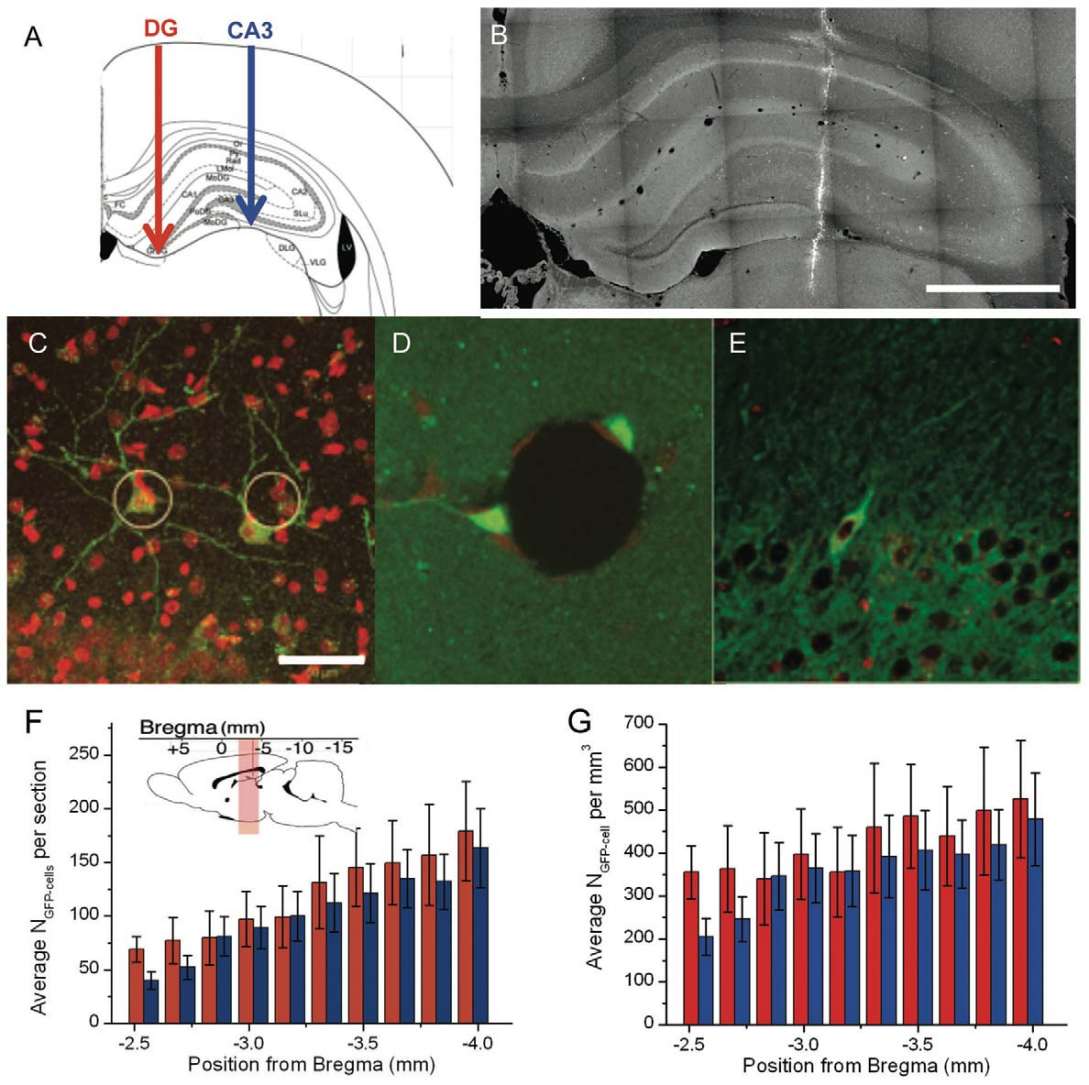
Transplanted neurons in the adult rat hippocampus. DIV 5 GFP-neurons were injected unilaterally into the right hippocampus of 6 weeks old rats using 45 µm bead carriers. **a**) Schematic representation showing the injection location in the dentate gyrus (DG), and in the CA3 region (CA3). After a week the animals were sacrificed and their brains were sliced and immuno-stained with GFP antibody (green) and with Nissl (red) a nuclear cell marker. **b**) Fluorescence microscopy image of a brain slice taken at the injection site (scale bar  =  1 mm). Fields extracted from a XYZ-tile scan of the hippocampus, −3.7 mm A/P from the bregma, showing the extent of the transplanted neuron implantation in **c**) the CA3 stratum lucidum layer. **d**) Cross-section of a bead carrying two GFP+ neurons sending processes into the hippocampus in a 150 µm thick slice. **e**) A rare GFP+ neuron found in the brain section after dissociation from 2D support prior to injection. Scale bar  =  50 µm. Anterio-posterior GFP+ neuron distribution for injections made at [AP] = −3.5 in the CA3 (blue) and in the DG (red). **f**) shows the average number of GFP+ neuron (N_GFP-cell_), and **g**) shows the average number of GFP-neuron (N_GFP-cell_) per mm^3^. Error bars represent the standard deviations for series of 10 animals.

Neurons grown on beads, as described above, were infected with a lentiviral vector driving GFP from a synapsin promoter at DIV 2. Media was changed at DIV 4 to clear unbound viral particles, and cells were checked for GFP expression. At DIV 5 or 6 they were suspended in fresh media and injected stereotactically into the hippocampi of 6-week-old adult recipient animals. The injection was directed into the non-neurogenic CA3 region of the hippocampus previously used in successful homotopic transplantation in temporal lobe epilepsy animal model (**Fig. 2a, b** **;** for details see **Methods** section). The procedure was performed on 12 recipient animals, in three rounds of surgery, each with its own preparation of bead-borne neurons. One week after injection, the animals were sacrificed, and brain slices were imaged to assess cell transfer (**Fig. 2**). We found single GFP-positive (GFP+) neurons scattered across the hippocampus, and irrespective of the mediolateral or anteroposterior position of the injection (see **Supplementary Fig. S3 and Fig. S4**). When found, GFP+ cell aggregates were located only at the site of the injection. Whereas most cells had less than 5 branches (see **supplementary Fig. S5 a–c**), the transplanted cells in the hilus and SLu region had long processes, which extended into the host tissue, so that they resembled the morphology of the host neurons (**Fig. 2c–e**). In rare instances, cells were found associated with their carrier beads (**Fig. 2d**). In stark contrast, in all three animals that were injected with neurons that were dissociated from a standard 2D surface of a culture dish, rare dimly fluorescent cells could be detected. In most sections did not have GFP+ cells. Occasionally in the vicinity of the injection site we found 1 to 5 cells with a GFP-signal at least twice above background (**Fig. 2e**), suggesting a low rate of cell survival and/or poor cell health.

### Influence of the injection location toward neuron insertion

Local cues at the injection site can influence transplanted cell distribution and integration. For instance, NSCs transplanted into the CA3 that reach the dentate gyrus (DG) of the hippocampus — one of the few neurogenic areas of the brain— have been reported to adopt the cell fates of the sub-granular zone (SGZ) stem cells and to continue proliferating [31]. To evaluate the influence of the injection location on implantation of bead-borne differentiated neurons, we carried out additional transplantations into different mediolateral coordinates that targeted both the pyramidal CA3 region and the DG region (10 animals, 2 surgeries, 2 cell preparations). We found no significant difference in the anteroposterior (AP) distribution of the implanted neurons between injections into CA3 vs. into the DG (**Fig. 2f**). The average number of GFP+ neurons per unit volume across the hippocampus also remained the same (**Fig. 2g**). On average the bead-borne neuronal transplantations yielded 4,560±660 (n = 10) GFP+ neurons per injection (i.e. per brain hemisphere) corresponding to a survival rate of 76%±11% as a maximum of 120 beads carrying about 50 cells were injected. In contrast, only a small percentage of DIV5 neurons grown on 2D support survived the dissociation step prior to transplantation. At most we counted 5 GFP+ cells in section around the injection track whereas we would typically count more than 100 GFP+ cells at similar [AP] location in brain transplanted with cells on carriers, and we did not find cells elsewhere.

### Localization of transplanted neurons to areas of the hippocampus

**Figure 3:**
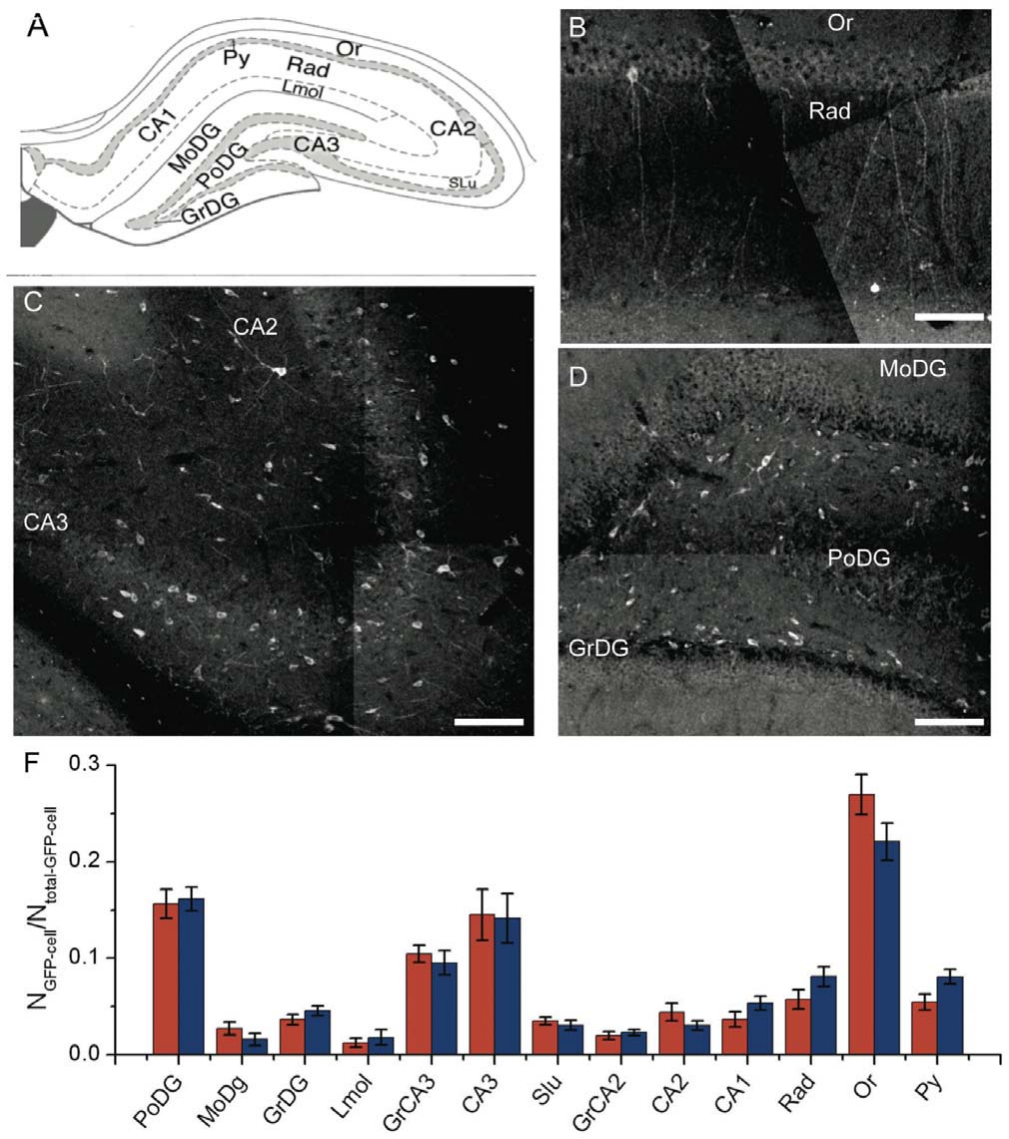
Influence of the injection position on the distribution of the implanted GFP-neurons. **a)** Schematic representation of the different hippocampus sub-regions. CA1-field SLu- stratum lucidum, Rad- radiatum layer of the hippocampus, PoDG- polymorph layer of the dentate gyrus, GrDG- granular layer of the dentate gyrus, MoDG- molecular layer of the dentate gyrus, LMol- lacunosum moleculare layer of the hippocampus, Py - pyramidal cell layer of the hippocampus, and Or -oriens layer of the hippocampus. Confocal microscopy images extracted from xyz-tile acquisitions showing GFP+ neuron implantation through out the hippocampus: **b**) shows the radiatum layer, **c**) the stratum lucidum of the CA3, **d**) part of the dentate gyrus. **f**) Fraction of the total GFP+ cells found in each region for injections in the CA3 (blue) and in the DG (red). Error bars represent the standard deviations for series of 10 animals. Scale bars  =  100 µm.

We also examined the partitioning of GFP+ neurons within the different regions of the hippocampus (**Fig. 3a**). In each region most of the transplanted cells exhibited extended processes into the host tissue and adopted a morphology expected for that subregion. For example, in the granular pyramidal sublayer (Py) they oriented their cell bodies parallel to the neighboring cells and projected process into the stratum radiatum (Rad) (**Fig. 3b**). In the CA2 region they exhibited the characteristic star-like branching (**Fig. 3c**). And in the DG the implanted cells primarily aligned themselves along the SGZ, and thus perpendicular to the cellular processes of the DG, while a small fraction inserted in the granular layer (**Fig. 3d**). However, we cannot tell if GFP+ cells projected to the expected region for their location because our brain sections were too thin for long distance tracing of neuronal projections. The distribution of the transplanted cells was the same for the injection into the DG and CA3, and the number of cells in each region scaled with the area of the brain section occupied by the sub-layer considered (**Fig. 3f**). The distribution may reflect either or both the mixture of identities of the transplanted neurons and the responsiveness to the local environment.

### Functional integration of the transplanted neurons

To determine whether the transplanted neurons made active synaptic contacts into the host circuit, we transplanted neurons expressing both GFP and the light-gated glutamate receptor (LiGluR6), which contains an introduced cysteine at position 439 that serves as an attachment site for the photo-switched tethered glutamate molecule MAG-1 [41], [42], [43]. This construct enables the remote excitation with UV-light of the neurons expressing LiGluR6 specifically without having to perform delicate electrophysiology recording. The activation is specific, as neurons labeled with MAG-1 but that do not express LiGluR6 are not activated by illumination at 390 nm [41]. A week after the transplantation of the GFP/LiGluR6-expressing bead-borne neurons, the recipient animals were sacrificed, and 210 µm thick hippocampal slices were prepared (**Methods**). The slices were incubated in artificial cerebrospinal fluid (ACSF) and labeled first with MAG-1 and then with Rhod-2, a fluorescent calcium indicator. MAG-1 selectively confers optical excitation only onto neurons expressing LiGluR6 [36], [42], whereas Rhod-2 loaded into all of the cells in the slice, enabling us to use confocal Ca^2+^ imaging to monitor neuronal activity in both the host neurons and the bead-borne transplanted neurons.

**Figure 4:**
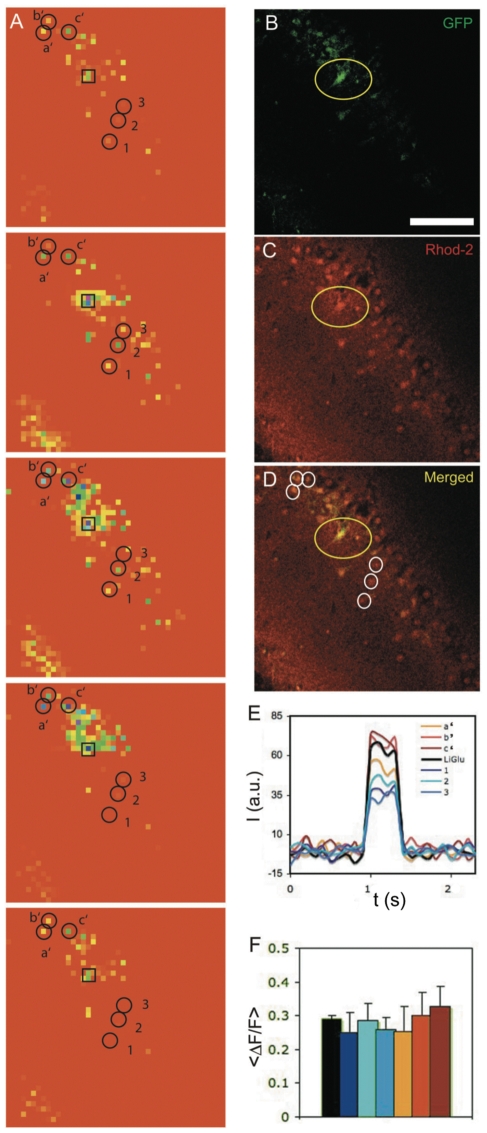
Functional integration of transplanted neurons. **a**) Live confocal imaging of calcium response in a hippocampal slice containing a transplanted LiGluR6 neuron expressing GFP **b**) and labeled with a calcium indicator, Rhod-2 **c**). Panel **d**) shows an overlay of both channels. Scale bar  = 100 µm. LiGluR6 cell was stimulated by short exposure to 390nm light for a short period of time and we recorded the calcium response of the surrounding neurons. **a**) shows calcium variation of individual cells (single pixel) after binning (3x3) and subtraction of the fluorescence background. Response was color-coded using a rainbow scale. Corresponding fluorescence intensity changes during UV stimulation are shown in panel **e**). All neurons in the slice responded to the stimulation indicating that the transplanted cell has made functional connections with the surrounding neurons. For 6 neurons distributed above (labeled *a b c*) and below (labeled *1 2 3*) the transplanted cell we calculated ΔF/F for seven UV stimulations **f**). ΔF/F of the LiGluR6 neuron remains around 30% (+/−2.5%). (a*bc*) neurons have, in average, higher ΔF/F than the stimulated neurons with significant variations from one exposure to the next, while (1 2 3) neurons have, in average, a smaller ΔF/F.

Illumination at 390 nm (using a frequency doubled 780 nm pulsed laser) was used to activate LiGluR6, and illumination at 543 nm was used to turn off its activity. The illumination at 390 nm reliably triggered a rise in Ca^2+^ in the GFP/LiGluR6 transplanted neurons in the DG region (**Fig. 4a–c**), and the illumination at 543 nm turned this activity off, thereby resulting in a return to resting Ca^2+^ levels (**Fig. 4e** **, black line**), results that are consistent with the excitation by LiGluR6 of the transplanted cells. Ca^2+^ imaging of the GFP-negative host neurons surrounding the GFP+ transplanted neurons revealed that they too were activated by illumination at 390 nm and deactivated at 543 nm **(** **Fig. 4e** **, colored lines,** and see **Supplementary movie S1**), even though they did not express LiGluR6. Thus, the Ca^2+^ responses observed in the GFP-negative host neurons around the transplanted GFP/LiGluR6-positive neurons suggest that the axons of the transplanted LiGluR6-expressing neurons make functional synapses with the dendrites of host DG and CA3 neurons. We calculated ΔF/F for 6 neurons (**Fig. 4a** **, circles**) distributed around a single transplanted neuron for a train of seven UV stimulations (**Fig. 4f**). Whereas the ΔF/F of the directly stimulated transplanted LiGluR6 neuron had little variability from one light pulse to the next, the host neuron responses were more variable from neuron to neuron and from pulse to pulse, consistent with their activation being mediated via heterogeneous synaptic connections. Host neurons that were farther from the transplanted neurons did not respond to the light pulses, suggesting that after one week, the transplanted neurons have a limited reach and can only produce strong enough synaptic excitation to nearby neurons to produce detectable rises in Ca^2+^. We obtained response for half of the cells recorded (n_cell_ = 8). These results demonstrate that a fraction of bead-borne neurons become functionally integrated into the network of the host brain within one week following transplantation.

### Long-term integration of the transplanted neurons

**Figure 5:**
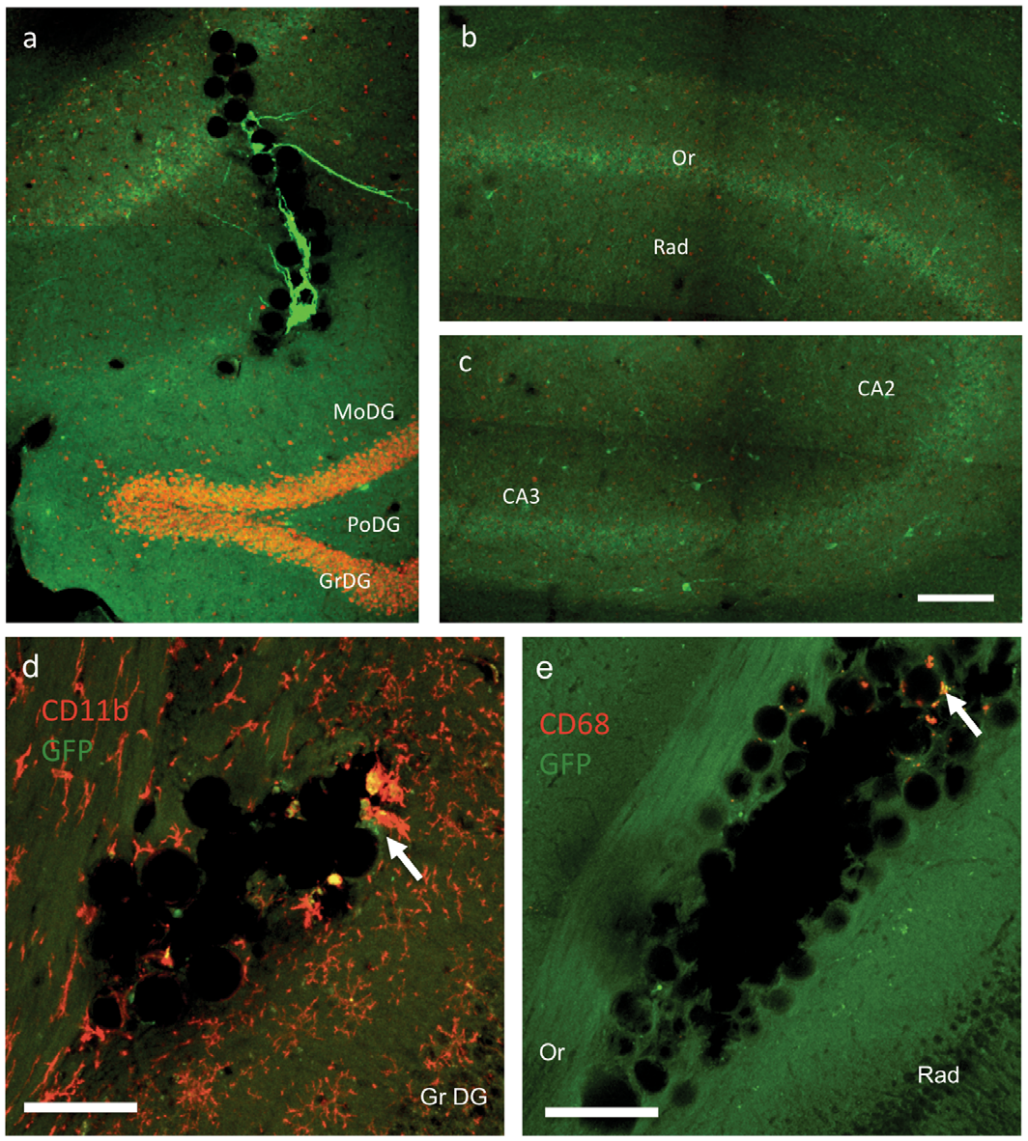
Long-term integration of the transplanted neurons. Confocal microscopy images extracted from xyz-tile acquisitions showing GFP+ neuron implantation throughout the hippocampus 24 weeks post-transplantation. **a**) shows beads at the injection site carrying GFP+ neurons which are projecting their processes in the host hippocampus, **b**) shows neurons in Or -oriens layer of the hippocampus sending out processes through the radiatum layer, and **c**) shows cells in the stratum lucidum of the CA3. Brain slices were stained with CD11b a marker for microglia cells (**d**), and CD68 a marker for macrophages (**e**). Confocal microscopy images 4 xy frames extracted from xyz-tile acquisitions showing glass bead cluster were projected in z. Increase in microglia cells and macrophages was associated with the presence of GFP+ cells without processes (arrows). Beads without cells were free of microglia and macrophages, suggesting that these cells were there to clear non-integrated GFP+ neurons. All scale bars  =  100 µm.

To determine if cells were transiently connected or fully integrated, we examined the partitioning of GFP+ neurons 6 months after transplantation. We found that transplanted cells still expressed GFP regardless of the injection location (**Fig. 5**). Carrier beads were still located at the injection site and baring bright GFP+ neurons which were sending long range processes from the Oriens region (Or) through the stratum radiatum (Rad) (**Fig. 5a**), the surrounding tissues remaining unaffected by the presence of the beads. No significant changes in transplanted cell distribution were observed, and neurons exhibited a healthy morphology (**Fig. 5b–c**). To assess the transplanted brains immune response we have carried out additional immuno-histochemistry on brain section one week and 24 weeks post-transplantation with a marker for microglia cells, CD11b, and a marker for macrophages, CD68. One week post-transplantation, we observed the typical tissue inflammation along the injection track (see **Supplementary Fig. S6**). After 24 weeks, we observed the injection track was cleared of inflation and we observed sporadic increase of CD11b positive cells and CD68 clearing GFP-positive cells that had remained on beads and failed to integrate (**Fig. 5d–e**).

## Discussion

We have shown that beads serve as neuronal growth supports for prolonged time periods and thus allow cells to mature into differentiated states. Culturing on the beads makes it possible to pre-engineer neurons *in vitro* to, for example, control cell differentiation and protein expression. It also provides an effective mean to by-pass the traditional cell supports, and the inherent damage induced upon dissociation from the surfaces, to obtain an injectable suspension for transplantation. Most of the bead-born neurons leave their carrier after the injection and disperse throughout the different regions of the host hippocampus, millimeters away from the injection location. Meanwhile, the presence of these non-degradable particles seems to be well tolerated. We did not observe an increase in tissue response due to injection of solid cell carriers compared to cell suspension. No macrophages or microglia cells were found on beads unless there were GFP+ neurons lacking processes that needed to be cleared. Beads remained at the injection site without any signs of adverse effects.

The transplanted neurons subsequently project extensive processes into the host tissue and form functional synaptic connections with the host cells. Although we cannot say statistically how many transplanted cells have made functional connections after one week, their long-term maintenance confirms they have functionally integrated into the host neuronal network. Further experiments will be needed to map GPF+ cells' neuronal projections, and to assess the strength of the synaptic connections made. Such approach holds the promise to positively impact diseases related to neuronal loss due to aging where intrinsic neurogenesis has almost completely receded [44], and diseases related to injuries such as ischemic strokes. However, the endogenous state of the injected brain greatly influences cell integration. Hence, to fully measure the therapeutic potential of our method, further functional studies in specific disease models will be needed.

Our method also provides an effective way to add new engineered differentiated neurons to the brain region from which they originate. Bead-born culture combines the benefits of the high integration observed with post-mitotic differentiated cells [45], with the high migration of stem cells [31] and without the side effects of seizure and tumor formation and with the additional benefit of a high survival rate. The long-range cell dispersion observed in non-injured young adult brain with low neurogenic and neurotrophic factors appears to be an intrinsic property of the bead-borne neurons, without significant variation between two injection sites suggesting that beads are more than just passive cell carriers.

Neurons from hippocampus, cortex, substantia nigra and cerebellum, which are routinely cultured *in vitro* [40], can be grown on carrier-beads using similar surface treatment, broadening the range of neuron types available for transplantation. Furthermore, preliminary experiments with soft nondegradable hydrogel particles suggest that this approach can be extended successfully to biodegradable materials. Combined with molecular engineering and the ease of bead sorting using microfluidics devices, this system provides a unique tool to specify the properties of the transplanted cells, either mature neurons or even cultures of differentiated neural stem cells where pre-sorting could be conducted to obtain properly differentiated neurons for transplantation. The method could therefore have application for *in vivo* developmental studies of interactions between a neuron and its nearby partners, as well as further pre-clinical studies for a broad range of disorders involving the loss of a specific type of neurons.

## Methods

### Neuron culture on silica bead preparation

Borosilicate Glass Spheres (MO-SCI Specialty Products, Rolla, MO) were sterilized in an ethanol solution overnight and dried under vacuum. The beads were then incubated in a borate buffer solution for one hour, before being left in PLL solution overnight. Beads were then removed from the PLL solution, and left to dry in a biosafety cabinet before being extensively washed in sterile water to remove unbound PLL. Hippocampi were removed from embryonic day 18 (E18) rats and treated with 0.3% trypsin for 15–20 min at 37°C, followed by washing and trituration. Poly-lysine-coated glass beads were distributed in a 12-well plate to cover about half of the available surface. For transplantation, dissociated cells were plated at 100,000 cells per well, leading to about 20 to 50 cells per 45 µm bead, and cultured in neurobasal medium supplemented with 2 mM Glutamax, 100 unit/mL Penicillin, 100 µg/mL Streptomycin, and 2% B-27. At DIV 1, the cell medium was conditioned with media harvested from a two-week-old 2D neuronal culture. Whereas ara-C is necessary to prevent glia cell proliferation in a long-term culture (∼2 weeks), it was not required for the five-days cultures used for transplant experiments. We observed that in the absence of ara-C, the number of glia cells present at DIV 5 was about 13% and did not affect the outcome of the transplantation (see **Supplementary Fig. S2** and **Table S1**). Hence we stopped adding ara-C to the culture medium for the last two transplantation series.

For long term tracing of the transplanted neurons, our hippocampal cultures were infected, once they had successfully settled on the beads at DIV 2, with a lentiviral vector that drives GFP expression from a synapsin promoter to restrict its expression to neurons only. The media was changed at DIV 4 to eliminate traces of unbound viral particles. At this point, the culture is considered free of infection particles (biosafety level 1). To enable remote excitation of the transplanted neurons with UV-light, our hippocampal culture was infected with adeno-associated viral vector driving LiGluR6 under a synapsin promoter following the same procedure as for the GFP lentiviral vector: infection at DIV 2, media change at DIV 4, injection at DIV 5 or 6.

### Cell dissociation from 2D in vitro culture

After dissection of E18 hippocampi part of cells was seeded on PLL coated coverslips at 75,000 cells per cm^2^. After five days *in vitro* culture they were lifted off the surface using a 5 minutes wash with HBSS without Ca^2+^ and Mg^2+^, followed by one minute incubation at 37°C in trypsin, and a mechanical shear from the surface using fresh medium. The resulting cell suspension was pelleted and resuspended in 100 µl medium to obtain comparable cell concentration in 2 µl as for the cell-carrier transplantation experiments.

### Immuno-staining

Anti-alpha-Tubulin (mouse; 1∶200), anti-GAD67 (rabbit; 1∶500), anti-GFAP (rabbit; 1∶1000), anti-Nestin (mouse; 1∶2000), anti-NeuN (mouse; 1∶500), anti-Prox-1 (rabbit; 1∶200) and anti-Synapsin-I (rabbit; 1∶1000) and anti-musashi (rabbit; 1∶200) antibodies were purchased from Chemicon International (Temecula, CA). Anti-Smi-312 (mouse; 1∶1000) and anti-Tuj-1 (rabbit; 1∶1000) were obtained from Covance (Berkeley, CA). Anti-CD11b (mouse; 1∶500), anti-CD68 (mouse; 1∶150), and anti-Sox2 (mouse; 1∶500) were purchased from Millipore. Anti-GFP (mouse; 1∶600) and Nissl-red (1∶100) were purchased from Invitrogen.

### *In vitro* cell culture composition

Beads were immobile during cell seeding, hence cells preferentially attached to the top hemispheres of the beads, leading to an asymmetric cell distribution on most of the beads. In a conditioned medium, we observed that a small number of cells undergo division leading to an increase in cell population and the formation of cell mass prone to detach under mechanical shear (see **Fig. 1g–i**). Cells appeared healthy with normal nuclear DAPI stain, and no detectable microglia cell population. We characterized by immuno-staining the cell population present at DIV 5-6 which corresponds to our harvesting time for transplantation. Results are summarized in **Supplementary Table S1**. We counted that 70.4% of the cells are NeuN positive, a nuclear marker for mature neurons, 13.2% are positive for GFAP, an astrocyte cell marker, whereas 16.1% are still positive for nestin, 5.6% are positive for musashi, and 4.8% are positive for sox2. We also determined that 10.7% of the NeuN positive cells are positive for GAD67 an inhibitory neuronal cell marker (see **Supplementary Fig. S2a–c**).

### Animal surgeries and histology

DIV 5 neurons cultures on 45 µm beads were stereotaxically injected into the hippocampus of the brain of adult female Fischer 344 rats (150 g, 6 weeks old) using Paxinos and Watson adult rat brain atlas coordinates in millimeters: (anteroposterior [AP], -3.5; mediolateral [ML], ±3.0; dorsoventral [DV], –3.9) for CA3 injections and (anterior-posterior [AP], −3.5; mediolateral [ML], ±1.15; dorsoventral [DV], –4.1) for DG injections. The animals were deeply anesthetized with a mixture of ketamine (90 mg/kg) and xylazine (10 mg/kg) before injection, and 0.5 µl of the bead suspension (approximately 70 beads on average), with a measured maximum of 120, was injected with a 26-gauge beveled needle mounted on a 2 µl Hamilton syringe. Brains were excised one week post-injection to assess the implantation of injected neurons by quantifying GFP expression (*n = 6 animals per series*). Animals were transcardially perfused with 0.9% (w/v) saline, followed by 4% paraformaldehyde in phosphate buffered saline. After retrieval, brains were post-fixed by immersion in 4% paraformaldehyde overnight at 4°C and subsequently stored in 30% sucrose for cryoprotection before sectioning.

Two techniques were used to make coronal sections. To document cell-bead implantation, we used a vibratome to prepare 100 µm thick sections and imaged the endogenous GFP signal on an inverted confocal microscope (Zeiss LSM 510 Axiovert 200) using a 20x air objective and 40x oil objective (1.3 N.A.). To establish the statistics of cell distribution throughout the hippocampus, we prepared sequential 45 µm thick sections for immunostaining using a cryomicrotome, and GFP expression was amplified with primary rabbit anti-GFP. Corresponding secondary antibodies (labeled with Alexa Fluor 488) were used to enhance detection. For nuclear staining, sections were stained with Nissl-red or a NeuN antibody. Full section imaging was performed on a Leica SP5-MP inverted microscope with a 20x oil objective (0.7 N.A.). Laser power, photomultiplier gain and filter sets were selected to minimize bleaching and bleed-through between channels (Alexa488 and GFP: ex 488nm, em 500-550nm; Cy3: ex 543nm, em 565-615nm; Alexa647: ex 633nm, em 650-700nm). For each frame, z stacks were acquired (8 to 10 images 5 µm apart); Frames were then tiled together using Leica's auto-stitching routine to visualize the entire coronal section and thus facilitate the identification of the different sub-layers of the hippocampus. We used the 5th edition of the Paxinos & Watson rat brain atlas to assign the cell location. Raw images were converted to tiff format with Fiji, an ImageJ based software, which includes additional plug-ins. The number of GFP+ cells was determined by a direct cell count for every fourth coronal section of each brain. Cell counting and region assignment were performed manually using “Cell counter” plug-in in Fiji to record cell coordinates and the corresponding hippocampal region. Acquired data was then exported into a tab separated file format which includes x,y,z coordinates, a region field identification tag of the counted cells as well as slice number and the slice absolute position with respect to the injection point. The resulting file was imported into a MySQL database to facilitate easy manipulation of the data. Custom script was developed in Python to access the information stored in MySQL, to parse the transplanted cell population, and to produce cell distribution statistics for all the rats in our study.

Animal protocols were approved by the University of California, Berkeley (UCB) Animal Care and Use Committee and by the Technische Universität Dresden and conducted in accordance with National Institutes of Health (NIH, Bethesda, MD) guidelines and the Regierungspräsidium Dresden.

### Cell survival analysis

The number of injected cells depends on the number of beads injected and the number of cells per bead. To establish how many beads on average were injected we counted the number of beads present in the injection volume. Glass beads were dense and accumulated at the tip of the needle and were released first; hence to maximize the number of beads we loaded the syringe with a larger volume than the injected quantity. The injection syringe was loaded with the bead suspension and the injected volume was released on a coverglass to enable the imaging of the droplet content. In 35 trials, we found that on average we could release 35 beads (SED = 32 with a peak at 120) in 0.5 µl after loading 2 µl in the syringe. Based on the seeding conditions and cell counting in DIV 5-6 fixed samples we estimated that the beads carried between 20 to 50 cells. A more precise statistical number of cells per bead could not be obtained because many cells detached from their carrier during the washing steps required and remained in the sample as floating aggregates. Hence, to estimate the number of cells injected, we considered the highest number of cells found on beads, 50, and multiplied it by the maximum of beads found in the injected volume, 120, leading to an upper bound of 6,000 injected cells.

### Brain slice preparation for functional activity

Rats were anaesthetized with halothane and killed by decapitation a week after the intracranial injection, in accordance with institutional guidelines. Horizontal midbrain slices (250-µm thick) were cut using a vibratome (Vibratome Company). Slices were prepared at 4–6°C in a solution containing 110 mM choline chloride, 2.5 mM KCl, 1.25 mM NaH_2_PO_4_, 0.5 mM CaCl_2_, 7 mM MgSO_4_, 26 mM NaHCO_3_, 25 mM glucose, 11.6 mM sodium ascorbate, and 3.1 mM sodium pyruvate. The slices were incubated in artificial cerebrospinal fluid (ACSF) containing 125 mM NaCl, 3 mM KCl, 2 mM CaCl_2_, 1 mM MgCl_2_, 1.25 mM NaH_2_PO_4_, 26 mM NaHCO_3_, and 10 mM glucose. The solutions were saturated with 95% medical air and 5% CO_2_.

### MAG labeling and illumination protocol

Conjugation of MAG-1 to iGluR6(L439C) in hippocampal neurons for optical switching experiments was based on a method described earlier [41]. MAG-1 compound was diluted to 25 µM in ACSF solution and pre-activated by UV light (365 nm) for 1 min to enhance conjugation by affinity labeling [42], [46]. Brain slices were incubated in the dark in 1 mL of the labeling solution for 15 min at 37°C. Subsequently, cells were loaded for 10-15 min with Rhod-2 (Invitrogen) 1 µlofa 5 µM in 20% pluronic acid stock in 1 mL of the labeling solution, and then washed three times with the ACSF solution. After a 15 min recovery period, the cultures were examined to confirm neuronal activity. The solutions were saturated with 95% O_2_ and 5% CO_2_.

Two brain sections from each rat were imaged on an inverted confocal microscope (Zeiss LSM 510 Axiovert 200) using a 40x oil objective (1.3 N.A.), and two GFP+ were recorded in each section. Neurons were illuminated with 390 nm light (frequency doubled 780 nm) to activate LiGluR6, and 543 nm illumination was used both to image Rhod-2 and to deactivate LiGluR6. Images were acquired at the rate of 8 frames per second.

## Supporting Information

Figure S1 **Mature neurons on the beads.** Neurons at DIV 14 in conditioned media with araC. Neuronal processes are stained with alpha-tubulin antibody (green); pre-synaptic terminals are stained with synapsin antibody (red). Processes can be seen crossing between beads to make synaptic contacts with neighboring neurons. Beads are 125 µm in diameter. Scale bar  =  100 µm.
(TIF)

Figure S2 **In vitro culture characterization.** E18 hippocampal neurons on 45 µm glass beads. Conditioned media was applied from DIV 1 on. Confocal microscopy z series are projected on the xy scanning plane. Cell composition of the culture was determined by immuno-cytochemistry. Cells were fixed at DIV 6 and stained with specific cell markers: (**a**) with NeuN (red), a specific nuclear marker for mature neurons, and GAD67 (green), a inhibitory neuronal cell marker, (**b**) with GFAP (green), an astrocyte cell marker, (**c**) with Nestin (green), a marker for neuronal stem cell, and (**d**) with the progenitor cell marker Sox2 (green) and Musashi (red), and imaged by confocal microscopy. Scale bars  =  50 µm. The total number of cells was established by counting cell nuclei stained with DAPI. Cells positive for these markers were then counted and the statistical results are summarized in the **table S1**.
(TIF)

Figure S3 **Anteroposterior distribution of the GFP-neurons.** Tile reconstruction of confocal XYZ imaging series of brain slices taken at different anterior-posterior location; −2.5 mm from the bregma, −3.5 mm, and −3.5 mm from the bregma. The images were obtained performing a maximum Z projection of 7 planes from confocal z section. Scale bar  =  1 mm.
(TIF)

Figure S4 **3D reconstruction of the GFP+ neurons distribution throughout the imaged brain.** Sequential brain slices were collected and every fourth slice was stained with GFP antibody to enhance the GFP signal. For all the stained sections, the number of GFP+ cells per brain slice was determined by a direct cell count. Cell counting and region assignment were performed manually using “Cell counter” plug-in in Fiji to record cell coordinates and the corresponding hippocampal region. Acquired data was then exported into a tab separated file format which includes x,y,z coordinates, region field identification tag of the counted cells as well as slice number and the slice absolute position with respect to the injection point. The resulting file was imported into a MySQL database to facilitate easy manipulation of the data. This graph represents the XYZ coordinates of the GFP+ neurons counted in the left hemisphere of one rat that was injected in the DG region. Cell populations were color-coded based on their location: cells in CA1 (purple circles), in CA2 (red down triangles), in CA3 (green stars), in DG (purples triangles), and in Or-Py (blue star).
(TIF)

Figure S5 **Transplanted cell morphology a week post injection (a-c) and 24 weeks later (d-f).** GFP+ cells found in different parts of the hippocampus were imaged at higher magnification. One week post injection, GFP+ cell next to the CA3 granular layer (**a**), hilus (**b**), subgranular layer of the dentate gyrus (**c**), showed a low number of branches. The highest degree of branching was observed in the Stratum Lucidum (SLu) region (**fig. 2c**) and in the Pyramidal tract (Py). It is worth noting that none of the GFP+ cells imaged in the Dentate Gyrus (DG) are positive for Prox-1, a characteristic marker of DG granular layer. After 24 weeks, GFP+ cells have developed an extensive arbor of neuronal processes. GFP+ cell above the Oriens layer (Or) (**d**), in the Or (**e**) exhibit an extensive branching. The large field of view of the CA2-CA3 region shows the mesh of oriented processes. The extent of this arbor is such that in 45 µm thick coronal sections, processes can rarely be traced back to the GFP+ neuron they originated from, suggesting that transplanted cells are successfully integrated. Scale bars  =  50 µm.
(TIF)

Figure S6 **Immune response.** DIV 5 GFP+ neurons were injected unilaterally into the right hippocampus of six-week-old rats, using 45 µm bead carriers. The animals were sacrificed after one week (**a–b**) and 24 weeks (**c–d**) and their brains were sliced and immuno-stained with CD11b antibody a microglia cell marker (**a, c**), and with CD68 antibody a macrophage marker (**b, d**). After one week, microglia cells and macrophages were distributed around the injection track (arrows). After 24 weeks, the microglia and macrophage cells found were associated with GFP+ cells without processes, suggesting that microglia and macrophage were clearing non-integrated cells. The same volume of DIV5 GFP+ cell suspension without carriers was injected unilaterally into the right hippocampus of six-week-old rats to evaluate tissue response to cell injection in absence of beads. The animals were sacrificed after one week their brains were sliced and immuno-stained with CD11b antibody a microglia cell marker (**e–f**). The stereotactic injection leads to tissue response along the injection track similar the one observed with cell-carriers suggesting that the presence of beads does not trigger additional tissue response. Scale bars  =  1mm.
(TIF)

Movie S1 **Live confocal imaging of calcium response in a hippocampal slice containing a transplanted LiGluR6 neuron expressing GFP.** LiGluR6 of the transplanted cells was excited by UV light and the movie shows calcium variation of individual cells (single pixel) after binning (3x3) and subtraction of the fluorescence background. The response was color-coded using a rainbow scale.
(MOV)

Table S1 **In vitro culture characterization.** Cell composition of the E18 hippocampal neurons on 45 µm glass beads was determined by immuno-cytochemistry. The total number of cells was established by counting cell nuclei stained with DAPI. Cells positive for NeuN, GAD67, GFAP, Nestin, Sox2, Musachi were then counted and the statistical results are summarized in table S1.
(TIF)

## References

[1] Selkoe DJ (1991). The molecular pathology of Alzheimer's disease.. Neuron.

[2] Arendt T, Henning D, Gray JA, Marchbanks R (1988). Loss of neurons in the rat basal forebrain cholinergic projection system after prolonged intake of ethanol.. Brain Res Bull.

[3] Hirsch E, Graybiel AM, Agid YA (1988). Melanized dopaminergic neurons are differentially susceptible to degeneration in Parkinson's disease.. Nature.

[4] Damier P, Hirsch EC, Agid Y, Graybiel AM (1999). The substantia nigra of the human brain. II. Patterns of loss of dopamine-containing neurons in Parkinson's disease.. Brain 122 (Pt.

[5] Damier P, Hirsch EC, Agid Y, Graybiel AM (1999). The substantia nigra of the human brain. I. Nigrosomes and the nigral matrix, a compartmental organization based on calbindin D(28K) immunohistochemistry.. Brain.

[6] Fearnley JM, Lees AJ (1991). Ageing and Parkinson's disease: substantia nigra regional selectivity.. Brain.

[7] Everitt BJ, Robbins TW (1997). Central cholinergic systems and cognition.. Annu Rev Psychol.

[8] Shepherd GM (1979). The synaptic organization of the brain..

[9] Borlongan CV, Saporta S, Poulos SG, Othberg A, Sanberg PR (1998). Viability and survival of hNT neurons determine degree of functional recovery in grafted ischemic rats.. Neuroreport.

[10] Saporta S, Borlongan CV, Sanberg PR (1999). Neural transplantation of human neuroteratocarcinoma (hNT) neurons into ischemic rats. A quantitative dose-response analysis of cell survival and behavioral recovery.. Neuroscience.

[11] Hurlbert MS, Gianani RI, Hutt C, Freed CR, Kaddis FG (1999). Neural transplantation of hNT neurons for Huntington's disease.. Cell Transplant.

[12] Zaman V, Shetty AK (2001). Fetal hippocampal CA3 cell grafts transplanted to lesioned CA3 region of the adult hippocampus exhibit long-term survival in a rat model of temporal lobe epilepsy.. Neurobiol Dis.

[13] Baker KA, Hong M, Sadi D, Mendez I (2000). Intrastriatal and Intranigral Grafting of hNT Neurons in the 6-OHDA Rat Model of Parkinson's Disease.. Experimental Neurology.

[14] Lindvall O, Hagell P (2000). Clinical observations after neural transplantation in Parkinson's disease.. Prog Brain Res.

[15] Redmond DE, Vinuela A, Kordower JH, Isacson O (2008). Influence of cell preparation and target location on the behavioral recovery after striatal transplantation of fetal dopaminergic neurons in a primate model of Parkinson's disease.. Neurobiol Dis.

[16] Freed CR, Breeze RE, Rosenberg NL, Schneck SA, Kriek E (1992). Survival of implanted fetal dopamine cells and neurologic improvement 12 to 46 months after transplantation for Parkinson's disease.. N Engl J Med.

[17] Dunnett SB (1995). Functional repair of striatal systems by neural transplants: evidence for circuit reconstruction.. Behav Brain Res.

[18] Sanberg PR, Borlongan CV, Koutouzis TK, Norgren RB, Cahill DW (1997). Human fetal striatal transplantation in an excitotoxic lesioned model of Huntington's disease.. Ann N Y Acad Sci.

[19] Shetty AK, Turner DA (1995). Enhanced cell survival in fetal hippocampal suspension transplants grafted to adult rat hippocampus following kainate lesions: a three-dimensional graft reconstruction study.. Neuroscience.

[20] Conti L, Reitano E, Cattaneo E (2006). Neural stem cell systems: diversities and properties after transplantation in animal models of diseases.. Brain Pathol.

[21] Kim M, Lee ST, Chu K, Kim SU (2008). Stem cell-based cell therapy for Huntington disease: a review.. Neuropathology.

[22] Kelly S, Bliss TM, Shah AK, Sun GH, Ma M (2004). Transplanted human fetal neural stem cells survive, migrate, and differentiate in ischemic rat cerebral cortex.. Proc Natl Acad Sci U S A.

[23] Bjorklund LM, Sanchez-Pernaute R, Chung S, Andersson T, Chen IY (2002). Embryonic stem cells develop into functional dopaminergic neurons after transplantation in a Parkinson rat model.. Proc Natl Acad Sci U S A.

[24] Kawasaki H, Mizuseki K, Nishikawa S, Kaneko S, Kuwana Y (2000). Induction of midbrain dopaminergic neurons from ES cells by stromal cell-derived inducing activity.. Neuron.

[25] Barberi T, Klivenyi P, Calingasan NY, Lee H, Kawamata H (2003). Neural subtype specification of fertilization and nuclear transfer embryonic stem cells and application in parkinsonian mice.. Nat Biotechnol.

[26] Kim JH, Auerbach JM, Rodriguez-Gomez JA, Velasco I, Gavin D (2002). Dopamine neurons derived from embryonic stem cells function in an animal model of Parkinson's disease.. Nature.

[27] Lindvall O, Kokaia Z (2006). Stem cells for the treatment of neurological disorders.. Nature.

[28] Kim SU, de Vellis J (2009). Stem cell-based cell therapy in neurological diseases: a review.. J Neurosci Res.

[29] Shetty AK, Hattiangady B (2007). Concise review: prospects of stem cell therapy for temporal lobe epilepsy.. Stem Cells.

[30] Naegele JR, Maisano X, Yang J, Royston S, Ribeiro E (2010). Recent advancements in stem cell and gene therapies for neurological disorders and intractable epilepsy.. Neuropharmacology.

[31] Carpentino JE, Hartman NW, Grabel LB, Naegele JR (2008). Region-specific differentiation of embryonic stem cell-derived neural progenitor transplants into the adult mouse hippocampus following seizures.. J Neurosci Res.

[32] Bibel M, Richter J, Lacroix E, Barde YA (2007). Generation of a defined and uniform population of CNS progenitors and neurons from mouse embryonic stem cells.. Nat Protoc.

[33] Moon SY, Park YB, Kim DS, Oh SK, Kim DW (2006). Generation, culture, and differentiation of human embryonic stem cells for therapeutic applications.. Mol Ther.

[34] Espinosa-Jeffrey A, Wakeman DR, Kim SU, Snyder EY, de Vellis J (2009). Culture system for rodent and human oligodendrocyte specification, lineage progression, and maturation.. Curr Protoc Stem Cell Biol Chapter 2: Unit 2D.

[35] Yang D, Zhang ZJ, Oldenburg M, Ayala M, Zhang SC (2008). Human embryonic stem cell-derived dopaminergic neurons reverse functional deficit in parkinsonian rats.. Stem Cells.

[36] Pautot S, Wyart C, Isacoff EY (2008). Colloid-guided assembly of oriented 3D neuronal networks.. Nat Methods.

[37] Turner DA, Shetty AK (2003). Clinical prospects for neural grafting therapy for hippocampal lesions and epilepsy.. Neurosurgery.

[38] Letourneau PC (1975). Possible roles for cell-to-substratum adhesion in neuronal morphogenesis.. Developmental Biology.

[39] Letourneau PC (1975). Cell-to-substratum adhesion and guidance of axonal elongation.. Developmental Biology.

[40] Banker G, Goslin K (1998). Culturing Nerve Cells..

[41] Szobota S, Gorostiza P, Del Bene F, W C, Fortin DL (2007). Remote control of neuronal activity with a light-gated glutamate receptor.. Neuron.

[42] Gorostiza P, Volgraf M, Numano R, Szobota S, Trauner D (2007). Mechanisms of photoswitch conjugation and light activation of an ionotropic glutamate receptor.. Proceedings of the National Academy of Sciences.

[43] Volgraf M, Gorostiza P, Numano R, Kramer RH, Isacoff EY (2006). Allosteric control of an ionotropic glutamate receptor with an optical switch.. Nature Chemical Biology.

[44] Ehninger D, Kempermann G (2008). Neurogenesis in the adult hippocampus.. Cell Tissue Res.

[45] Shetty AK, Zaman V, Turner DA (2000). Pattern of long-distance projections from fetal hippocampal field CA3 and CA1 cell grafts in lesioned CA3 of adult hippocampus follows intrinsic character of respective donor cells.. Neuroscience.

[46] Gorostiza P, Isacoff E (2007). Optical switches and triggers for the manipulation of ion channels and pores.. Mol Biosyst.

