# Supplementary figures and images for: Colloids as Mobile Substrates for the Implantation and Integration of Differentiated Neurons into the Mammalian Brain
Source: PLoS One. 2012 Jan 25;7(1):e30293. doi: 10.1371/journal.pone.0030293 (PMC3266246; doi:10.1371/journal.pone.0030293)

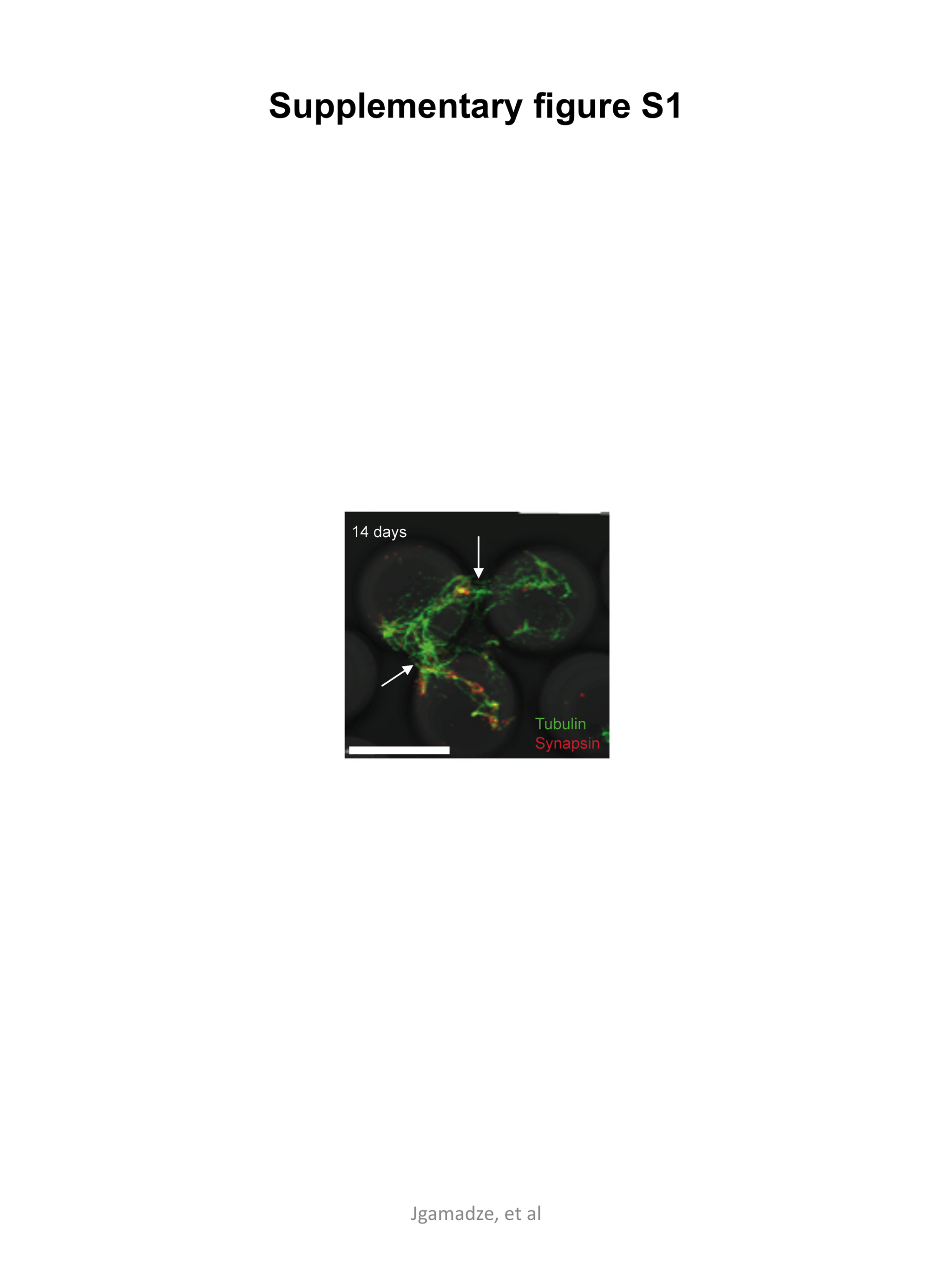

Supplement: Figure S1 — Mature neurons on the beads. Neurons at DIV 14 in conditioned media with araC. Neuronal processes are stained with alpha-tubulin antibody (green); pre-synaptic terminals are stained with synapsin antibody (red). Processes can be seen crossing between beads to make synaptic contacts with neighboring neurons. Beads are 125 µm in diameter. Scale bar = 100 µm. (TIF) [file pone.0030293.s001.tif]

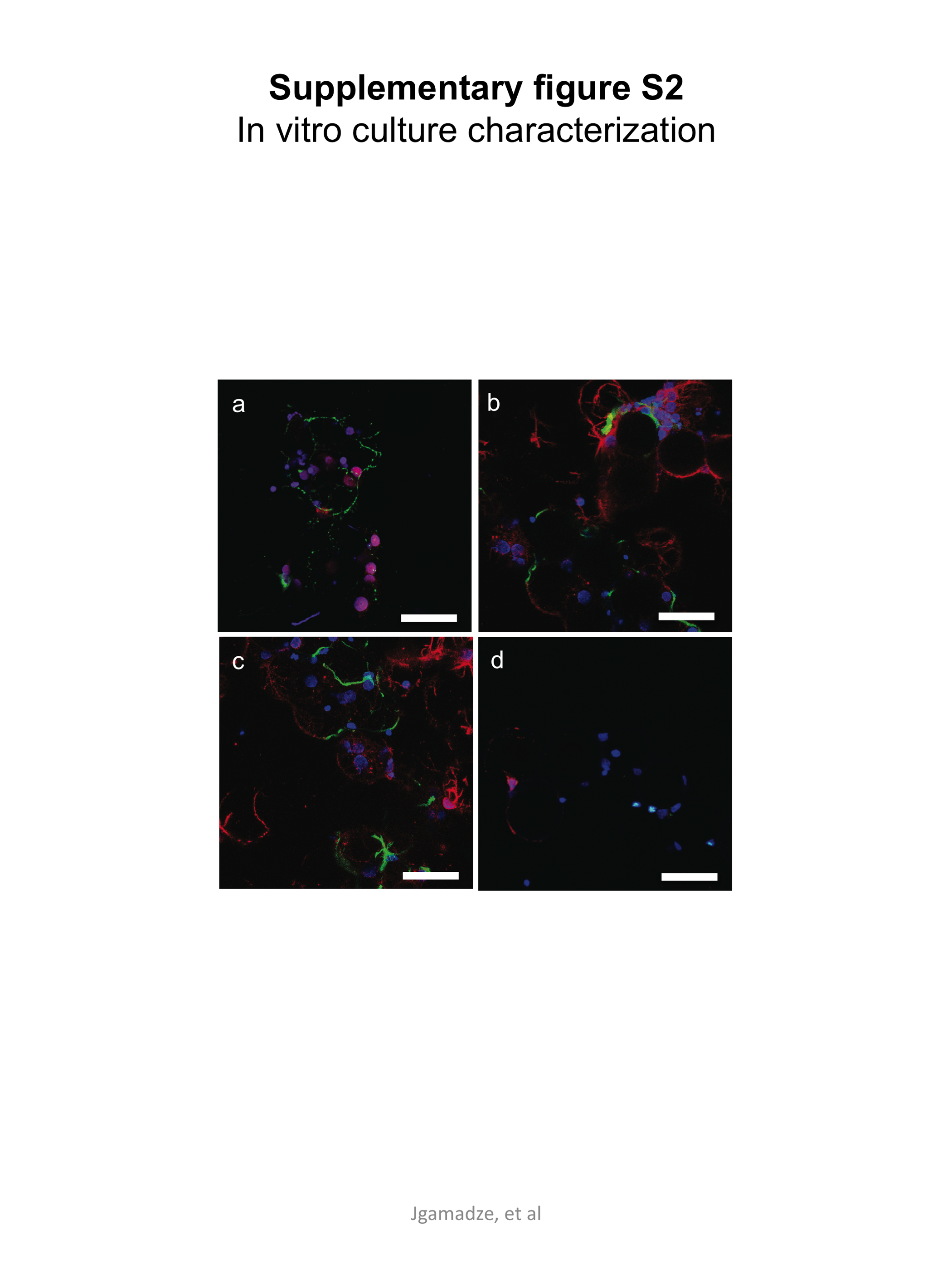

Supplement: Figure S2 — In vitro culture characterization. E18 hippocampal neurons on 45 µm glass beads. Conditioned media was applied from DIV 1 on. Confocal microscopy z series are projected on the xy scanning plane. Cell composition of the culture was determined by immuno-cytochemistry. Cells were fixed at DIV 6 and stained with specific cell markers: (a) with NeuN (red), a specific nuclear marker for mature neurons, and GAD67 (green), a inhibitory neuronal cell marker, (b) with GFAP (green), an astrocyte cell marker, (c) with Nestin (green), a marker for neuronal stem cell, and (d) with the progenitor cell marker Sox2 (green) and Musashi (red), and imaged by confocal microscopy. Scale bars = 50 µm. The total number of cells was established by counting cell nuclei stained with DAPI. Cells positive for these markers were then counted and the statistical results are summarized in the table S1. (TIF) [file pone.0030293.s002.tif]

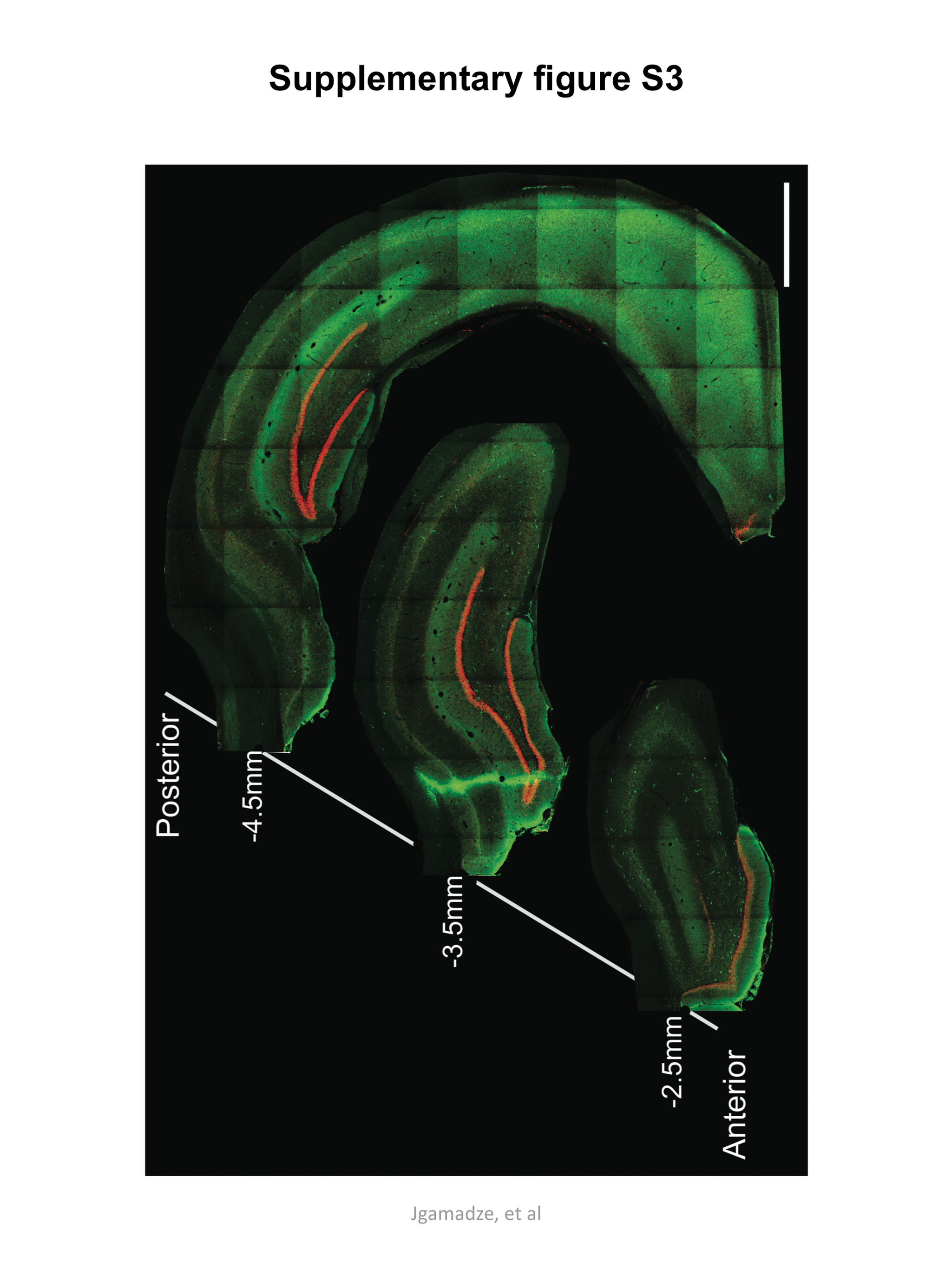

Supplement: Figure S3 — Anteroposterior distribution of the GFP-neurons. Tile reconstruction of confocal XYZ imaging series of brain slices taken at different anterior-posterior location; −2.5 mm from the bregma, −3.5 mm, and −3.5 mm from the bregma. The images were obtained performing a maximum Z projection of 7 planes from confocal z section. Scale bar = 1 mm. (TIF) [file pone.0030293.s003.tif]

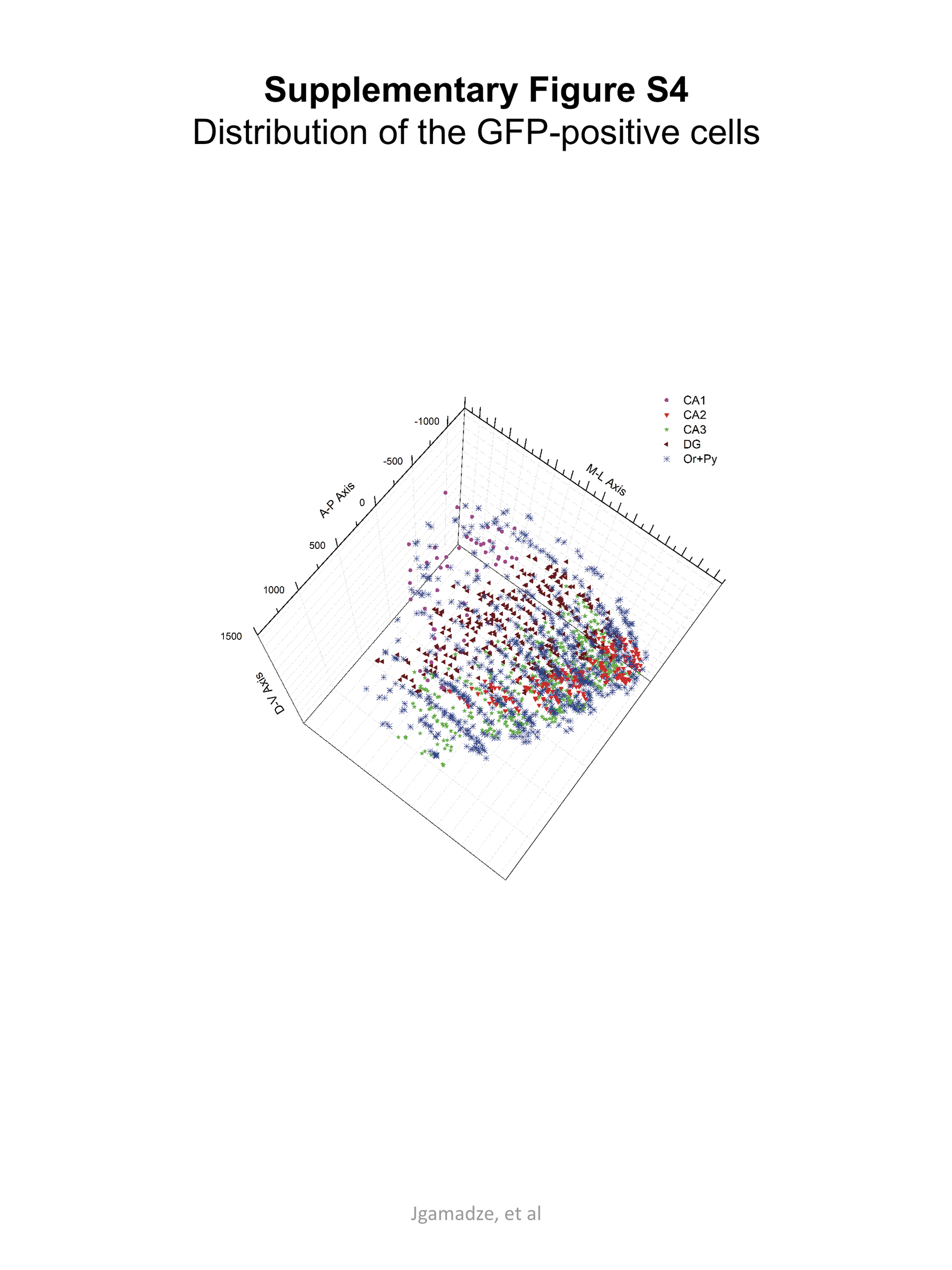

Supplement: Figure S4 — 3D reconstruction of the GFP+ neurons distribution throughout the imaged brain. Sequential brain slices were collected and every fourth slice was stained with GFP antibody to enhance the GFP signal. For all the stained sections, the number of GFP+ cells per brain slice was determined by a direct cell count. Cell counting and region assignment were performed manually using “Cell counter” plug-in in Fiji to record cell coordinates and the corresponding hippocampal region. Acquired data was then exported into a tab separated file format which includes x,y,z coordinates, region field identification tag of the counted cells as well as slice number and the slice absolute position with respect to the injection point. The resulting file was imported into a MySQL database to facilitate easy manipulation of the data. This graph represents the XYZ coordinates of the GFP+ neurons counted in the left hemisphere of one rat that was injected in the DG region. Cell populations were color-coded based on their location: cells in CA1 (purple circles), in CA2 (red down triangles), in CA3 (green stars), in DG (purples triangles), and in Or-Py (blue star). (TIF) [file pone.0030293.s004.tif]

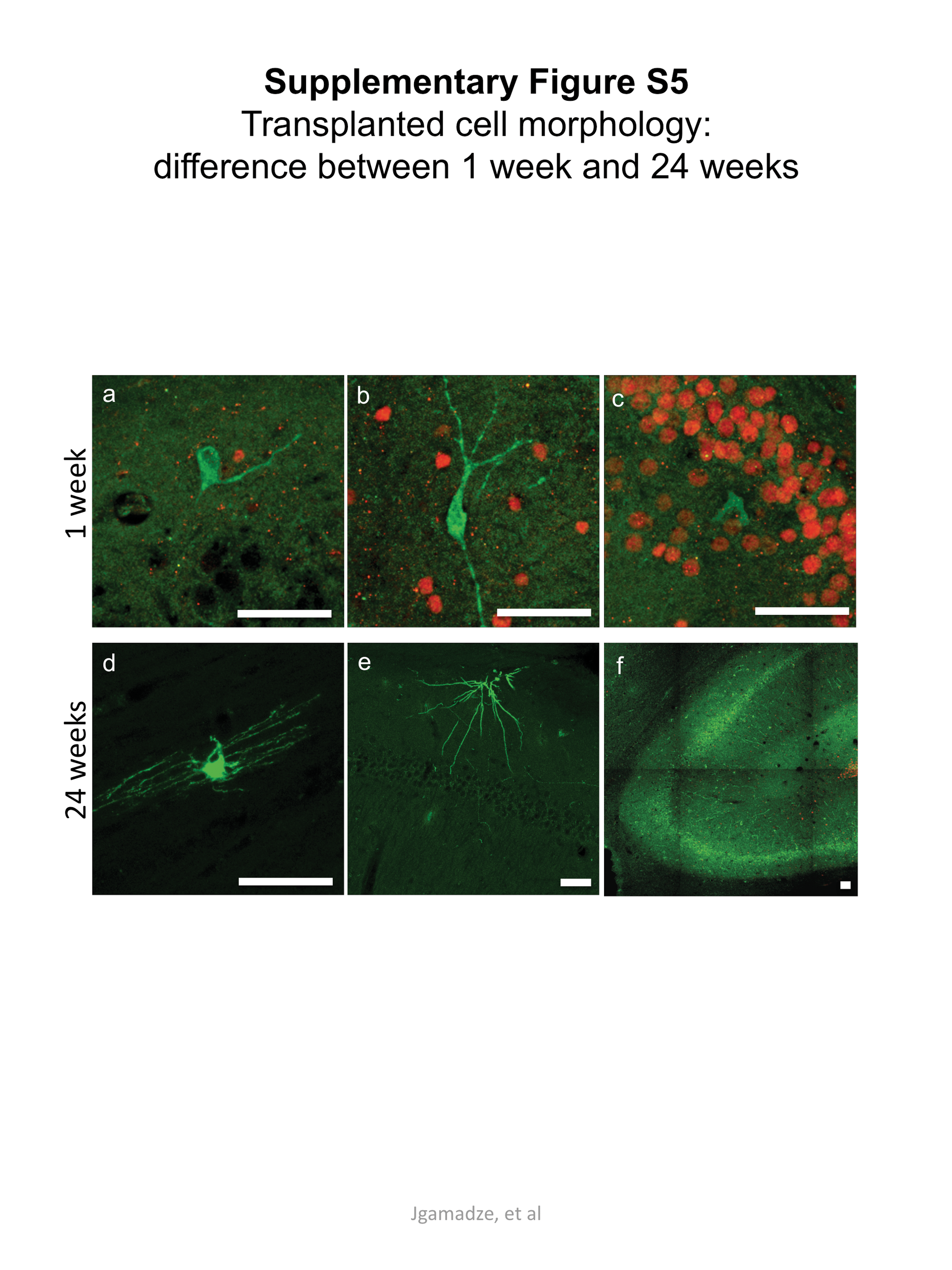

Supplement: Figure S5 — Transplanted cell morphology a week post injection (a-c) and 24 weeks later (d-f). GFP+ cells found in different parts of the hippocampus were imaged at higher magnification. One week post injection, GFP+ cell next to the CA3 granular layer (a), hilus (b), subgranular layer of the dentate gyrus (c), showed a low number of branches. The highest degree of branching was observed in the Stratum Lucidum (SLu) region ( fig. 2c ) and in the Pyramidal tract (Py). It is worth noting that none of the GFP+ cells imaged in the Dentate Gyrus (DG) are positive for Prox-1, a characteristic marker of DG granular layer. After 24 weeks, GFP+ cells have developed an extensive arbor of neuronal processes. GFP+ cell above the Oriens layer (Or) (d), in the Or (e) exhibit an extensive branching. The large field of view of the CA2-CA3 region shows the mesh of oriented processes. The extent of this arbor is such that in 45 µm thick coronal sections, processes can rarely be traced back to the GFP+ neuron they originated from, suggesting that transplanted cells are successfully integrated. Scale bars = 50 µm. (TIF) [file pone.0030293.s005.tif]

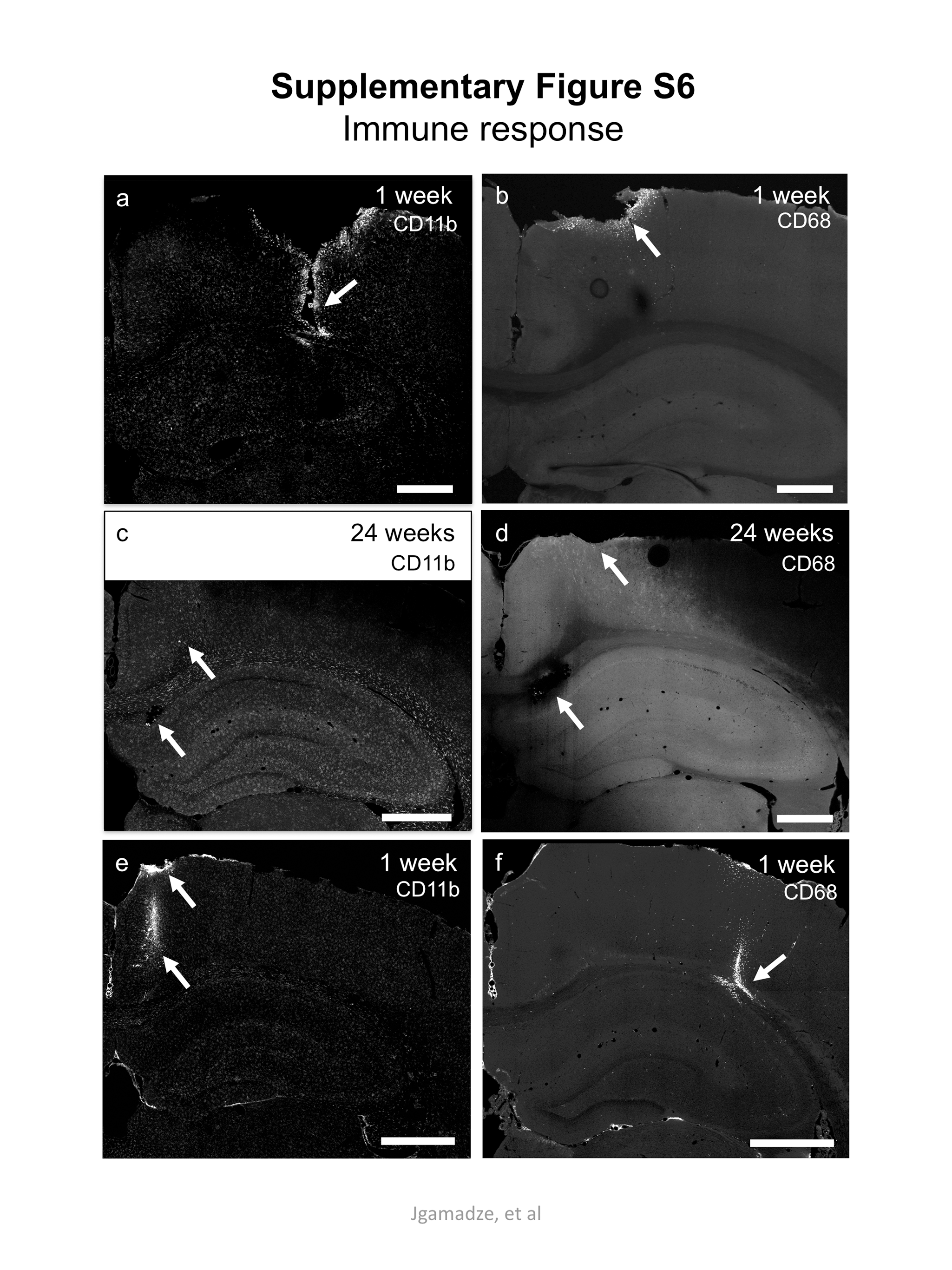

Supplement: Figure S6 — Immune response. DIV 5 GFP+ neurons were injected unilaterally into the right hippocampus of six-week-old rats, using 45 µm bead carriers. The animals were sacrificed after one week (a–b) and 24 weeks (c–d) and their brains were sliced and immuno-stained with CD11b antibody a microglia cell marker (a, c), and with CD68 antibody a macrophage marker (b, d). After one week, microglia cells and macrophages were distributed around the injection track (arrows). After 24 weeks, the microglia and macrophage cells found were associated with GFP+ cells without processes, suggesting that microglia and macrophage were clearing non-integrated cells. The same volume of DIV5 GFP+ cell suspension without carriers was injected unilaterally into the right hippocampus of six-week-old rats to evaluate tissue response to cell injection in absence of beads. The animals were sacrificed after one week their brains were sliced and immuno-stained with CD11b antibody a microglia cell marker (e–f). The stereotactic injection leads to tissue response along the injection track similar the one observed with cell-carriers suggesting that the presence of beads does not trigger additional tissue response. Scale bars = 1mm. (TIF) [file pone.0030293.s006.tif]

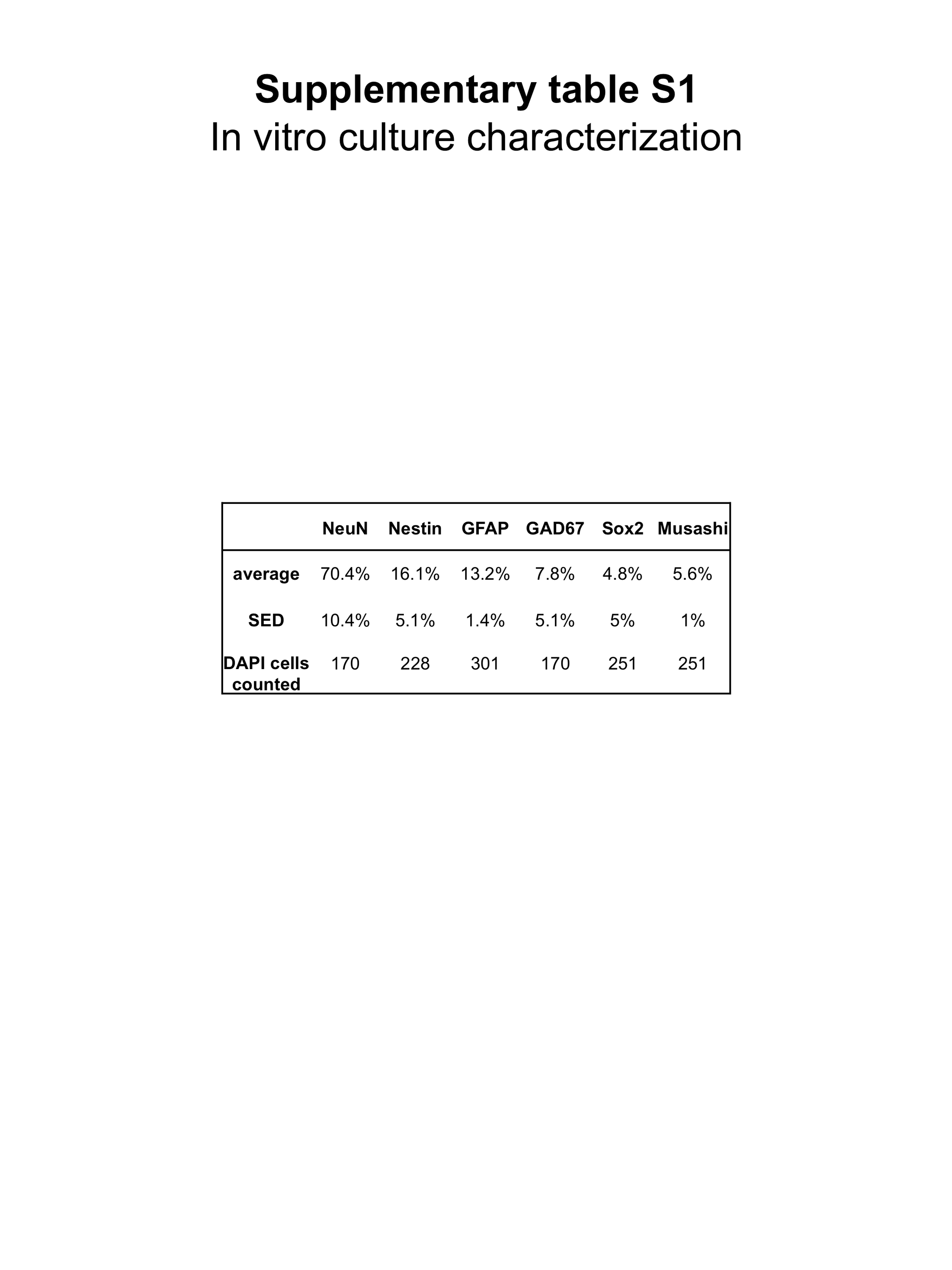

Supplement: Table S1 — In vitro culture characterization. Cell composition of the E18 hippocampal neurons on 45 µm glass beads was determined by immuno-cytochemistry. The total number of cells was established by counting cell nuclei stained with DAPI. Cells positive for NeuN, GAD67, GFAP, Nestin, Sox2, Musachi were then counted and the statistical results are summarized in table S1. (TIF) [file pone.0030293.s008.tif]
